# Competing risk models of stillbirth inform populations but not individuals

**DOI:** 10.1111/1471-0528.13943

**Published:** 2016-02-29

**Authors:** GCS Smith, IR White

**Affiliations:** ^1^ Department of Obstetrics and GynaecologyNIHR Cambridge Comprehensive Biomedical Research CentreUniversity of CambridgeCambridgeUK; ^2^ MRC Biostatistics UnitCambridge Institute of Public HealthCambridgeUK

Approaches to estimating the risk of antepartum stillbirth at a given week of gestational age have developed over the last 30 years. Previously, researchers related the number of antepartum stillbirths to the number of births at the given week. Then Yudkin et al. (*Lancet* 1987;1:1192–4) pointed out that all fetuses alive at the start of the given week were at risk of antepartum stillbirth, not just those that were born. Hence, the risk of antepartum stillbirth was calculated by the ratio of the number of stillbirths during the given week to the number of ongoing pregnancies at the start of the week. Next it was proposed that antepartum stillbirths should be analysed by time‐to‐event methods, which take into account censoring due to birth (Smith *Am J Obstet Gynecol* 2001;184:489–96). Now Naimi and Auger suggest that, when analysing cumulative risk of stillbirth using time‐to‐event methods, birth should be regarded not as censoring but as a ‘competing risk’ (*BJOG* 2016; 2016;123:1071–1074). There are, however, some important clinical issues that are not addressed by their analysis. First, they make no distinction between antepartum and intrapartum stillbirth. Second, it is not clear whether they have considered the fact that stillbirth of a dead twin may occur months after the actual in utero death. Third, 20–30% of births follow a medical or social decision (i.e. induction of labour or planned caesarean). The last of these points is a key issue in the analysis of post‐dates pregnancy and is best considered with an example of estimating stillbirth risk at 42 weeks.

A competing risk model calculates the risk of stillbirth during the 42nd week (using the UK definition of stillbirth) as the number of stillbirths in the 42nd week divided by the total number of pregnancies that were born at or after 24 weeks. Does it yield a valid answer? This depends on what the question was. The plot in Naimi and Auger's Figure 1 is the distribution of cumulative stillbirth risk across the range of gestational age when viewed at the start of the 24th week. The additional risk of stillbirth at 42 weeks is small, as most of the population will be delivered before then. However, what do we tell a woman who has reached the start of the 42nd week and wishes to know her risk of stillbirth if she postpones induction of labour until the start of the 43rd week? In this case we know that the woman did not deliver before 42 weeks. Hence, we need to calculate the subsequent risk of stillbirth *conditional* on the pregnancy reaching 42 weeks. The denominator is, therefore, all women who had an ongoing pregnancy at the start of the 42nd week, which is very different from the denominator in Naimi and Auger's analysis. Next, how do we calculate this risk of stillbirth in the 42nd week? We argue that neither simple time‐to‐event analysis nor competing risks analysis is wholly appropriate for a woman who declines induction of labour: rather, spontaneous labour should be treated as a competing risk, while induction of labour should be treated as censoring.

The approach described by Naimi and Auger is important. However, to use this approach to inform clinical decision making, for example, in estimating the effects of inducing labour at term or postterm, competing risk models should analyse the subsequent cumulative risk conditional on achieving a given week of gestational age, and distinguish between spontaneous and elective deliveries.

## Disclosure of interests

Full disclosure of interests available to view online as supporting information.
